# Comparison of endoplasmic reticulum stress and mitochondrial biogenesis responses after 12 weeks of treadmill running and ladder climbing exercises in the cardiac muscle of middle-aged obese rats

**DOI:** 10.1590/1414-431X20187508

**Published:** 2018-07-30

**Authors:** Kijin Kim, Nayoung Ahn, Suryun Jung

**Affiliations:** Department of Physical Education, Keimyung University, Daegu, Republic of Korea

**Keywords:** Exercise training, Mitochondrial biogenesis, Endoplasmic reticulum stress, Obesity, Cardiac muscle, Aging

## Abstract

The purpose of the present study was to compare the influence of aerobic exercise (AE) lasting 12 weeks to that of resistance exercise (RE) of the same duration on endoplasmic reticulum (ER) stress and mitochondrial biogenesis in the cardiac muscle of middle-aged obese rats. Obesity was induced in thirty 50-week-old male Sprague Dawley rats over 6 weeks by administration of a high-fat diet. The rats were then subjected to treadmill-running (AE) and ladder-climbing (RE) exercises 3 times per week for 12 weeks. Rats in the AE group showed significantly lower increases in body weight and intraperitoneal fat than those in the sedentary control (SC) group (P<0.05). The 12-week exercise regimes resulted in a significant increase in expression of mitochondrial biogenesis markers and levels of peroxisome proliferator-activated receptor gamma coactivator 1α in the cardiac muscle (P<0.05). Phosphorylation of PKR-like ER kinase, an ER stress marker, decreased significantly (P<0.05) after the exercise training. Although a trend for decreased C/EBP homologous protein (CHOP) expression was observed in both exercise groups, only the AE group had a statistically significant decrease (P<0.05). Levels of GRP78, an ER stress marker that protects cardiac muscle, did not significantly differ among the groups. Although only the AE group decreased body weight and fat mass, the two exercise regimes had similar effects on cardiac muscle with the exception of CHOP. Therefore, we suggest that both AE, which results in weight loss, and high-intensity RE, though not accompanied by weight loss, protect obese cardiac muscle effectively.

## Introduction

Cardiovascular diseases were responsible for 31.9% of deaths in the USA over the past 10 years, with 1 in 6 of these deaths being caused by coronary arterial diseases (1). In order to decrease the prevalence of cardiovascular diseases, risk factors, including hypertension, smoking, diabetes, dyslipidemia, obesity, and sedentary lifestyles, should be controlled. There is a social interest in urgently implementing efforts for the prevention and treatment of cardiovascular disease. In particular, obesity, an important cardiovascular disease risk factor, exerts negative influences on cardiac muscle through various pathways. According to recent research, activation of endoplasmic reticulum (ER) stress and chronic inflammatory responses in cardiac muscle is highly correlated with the occurrence of cardiovascular diseases, such as heart failure, atherosclerosis, and myocardial ischemia (2). Moreover, functional atrophy due to aging, accompanied by decreases in the contraction and mass of skeletal and cardiac muscle, affects cardiac function detrimentally (3). Age-related alterations in muscle cells are mainly caused by increased ER stress, altered metabolism, intracellular oxidative stress resulting from qualitative and quantitative changes in mitochondria, and modified Ca^2+^ signaling (4). Most notably, metabolic modifications induced by qualitative and quantitative mitochondrial changes have been reported to play vital roles in cardiac dysfunction and heart failure (5). Obesity is associated with mitochondrial dysfunction, including reduced mitochondrial O_2_ respiration and ATP production, increased mitochondrial ROS emission, and reduced ratio of mtDNA to nuclear DNA (6). Inflammation, mitochondrial dysfunction, and ER stress have been proposed to impair insulin sensitivity in diabetes (7), but the exact mechanisms by which the insulin signaling cascade becomes dysregulated heart function remain unknown. ER stress and mitochondrial biogenesis are inseparably related, and prevention of the former and promotion of the latter in the heart are important in maintaining cardiac function and preventing cardiac diseases (2).

Exercise training conducted at levels higher than the lactate threshold or over a long period of time represents the safest and most effective method of increasing mitochondrial biogenesis in muscle cells (8,9). Exercise training increases the number of mitochondria and oxidase activity (10) and protects the myocardium by increasing anti-oxidative enzyme activity (11). The ER is known to play a very important role in cardiac muscle cells. The signaling pathway in ER stress is mediated by three major ER stress sensors: PKR-like ER kinase (PERK), inositol requiring enzyme 1 (IRE1), and activating transcription factor 6 (ATF6). PERK is a key ER stress sensor and molecular mediator of the ER-mitochondria contact sites and is required to regulate inter-organellar cross-talk in ROS-induced cell death, a role not shared with other ER stress sensors, like IRE1. Therefore, PERK and pro-apoptotic C/EBP homologous protein (CHOP), a downstream of PERK, are key factors for understanding an association between ER stress and mitochondria malfunction caused by obesity (12). However, compared to knowledge of ER stress in other tissues (13), our understanding of this process in cardiac muscle is highly limited, and no clear correlation with mitochondria has yet been identified (14). Moreover, research concerning the effects of various types and methods of exercise on the expression of ER stress-related proteins is very scarce.

Therefore, in the present study, we sought to identify an exercise method capable of effectively preventing cardiovascular diseases through protection of the cardiac muscle. Specifically, we compared the effects between two 12-week exercise regimes (aerobic and resistance) on ER stress and mitochondrial biogenesis in the cardiac muscle of middle-aged rats with high-fat-diet-induced obesity.

## Material and Methods

### Subjects

Thirty 50-week old male Sprague Dawley rats were purchased from Hyochang Science (Republic of Korea) and habituated to their new environment for 1 week. Subsequently, obesity was induced by administering a high-fat diet (50% kcal from fat) for 6 weeks. After this period, each rat was randomly assigned to 1 of 3 treatment groups and subjected to 12 weeks of treatment while still receiving the high-fat diet. The body weight and food intake of all rats were measured to one decimal place 3 times per week at around 9 AM, after which, rats in the exercise groups underwent ladder climbing or treadmill running exercises. All rats were individually housed under a 12-h light/dark cycle at 24±1°C and in approximately 60% relative humidity. The present study was approved by the Animal Experiment Ethics Committee of Daegu Technopark Biohealth Center (BHCC-IACUC-2016-01). The treatment groups were as follows: sedentary control group (SC; n=10); resistance exercise group (RE; ladder climbing 3 days/week, n=10); aerobic exercise group: (AE; treadmill running 3 days/week, n=10).

### Diet formulation and treatment

The high-fat diet was formulated such that carbohydrate, fat, and protein comprised 30, 50, and 20%, respectively, of total caloric intake (Teklad diet, Envigo, USA). Vitamins (22 g/kg Teklad vitamin mix No. 40077, Envigo), minerals (51 g/kg Teklad mineral mix No. 170915), methionine (5 g/kg, Teklad Premier No. 10850), and choline chloride (1.3 g/kg) were also included (15). Rats had *ad libitum* access to double ion-exchanged water.

### Resistance exercise (RE)

The RE protocol used comprised ladder climbing and was developed by partially modifying the methods reported by Lee et al. (16). For the first week, the rats practiced climbing on a 1 m ladder at a 75° incline without weights on their tails to habituate them to the exercise. A cylinder containing weights was attached to the base of the tail with foam tape (3M Coban, 3M Deutschland GmbH, Germany) and a Velcro strap. Rats were positioned at the bottom of the climbing apparatus and motivated to climb the ladder by touching the tail. When the rats reached the top of the ladder, they were allowed to rest in a simulated home cage for 2 min. Subsequently, weights of 30-50% of their body weight were attached to the rats’ tails for ladder climbing training, and the weights and the number of repetitions were incrementally increased. From the second week until the completion of the exercise program, set 1 was conducted with weights of 70% of body weight, sets 2 and 3 with weights of 80% of body weight, sets 4 and 5 with weights of 90% of body weight, and sets 6-8 with weights of 100% of body weight. If a rat was able to climb the ladder with these loads, additional weights were placed in the cylinder in 30 g increments for each subsequent climb. Each set involved eight repetitions and was separated from the next by 2 min of rest. The exercise was conducted 3 times per week.

### Aerobic exercise (AE)

For the AE protocol, which comprised treadmill running, the methods reported by Koltai et al. (17) were used. Automated laboratory animal treadmills (Quinton Instruments, USA) were employed. For the first week, the rats underwent 5 min of exercise at a speed of 10 m/min on an incline of 0° for adaptation. Rats were first introduced to treadmill running for 3 days. The speed and incline of the treadmill were increased incrementally from the second week onwards, and then rats were trained for 30 min daily at a running speed of 10 m/min, on a 5% incline. The running speed and duration of the daily exercise sessions were then gradually increased to 60% of the animals' VO_2max_, on the last week of the 12-week training program. The rats ran at an intensity of 60% VO_2max_, corresponding to 22 m/min for 30 min. The intensity of the exercise program was carefully matched in the aerobic training group by VO_2max_ data, as described previously (17). This intensity was maintained until the completion of the experiment. AE training was conducted 3 times per week for 12 weeks. Although we set the exercise duration as 30 min, energy expenditure was not similarly controlled between RE training and AE training.

### Tissue extraction

After 12 weeks of treatment, the rats were allowed to rest for 48 h to avoid the “last-bout” effect. Subsequently, they were fasted for 12 h and anesthetized with Zoletil 50 (10 mg/kg body weight; Virbac, South Korea) and 2% Rompun (0.04 mL/kg; Bayer, South Korea). First, the abdominal cavity was opened to collect blood from the abdominal artery. The heart was then perfused with phosphate-buffered saline to remove blood, before being resected. After removal of the water, the heart was weighed. The clipped left ventricle was flash frozen and stored at -80°C until analysis. Intraperitoneal fat (epididymal, mesenteric, and retroperitoneal fat pads) was subsequently extracted and weighed.

### Tissue analysis

The extracted heart was homogenized in an ice-cold buffer containing 250 mM sucrose, 10 mM HEPES/1 mM EDTA (pH 7.4), 1 mM Pefabloc (Roche, Switzerland), 1 mM EDTA, 1 mM NaF, 1 μg/mL aprotinin, 1 μg/mL leupeptin, 1 μg/mL pepstatin, 0.1 mM bpV(phen), and 2 mg/mL glycerophosphate. The homogenized sample was freezed-thawed 3 times and centrifuged at 700 *g* for 10 min at 4°C. The supernatant was collected and proteins were quantified with a Bradford (18) assay. Samples were mixed with Laemmli buffer, loaded onto an SDS-polyacrylamide gel, and electrophoresed. Proteins were then transferred onto a nitrocellulose membrane, which was subsequently blocked for 60 min with 5% nonfat dry milk in TBS+0.1% Tween 10 (TBST; pH 7.5). After being washed with TBST, the membrane was incubated overnight with a primary antibody against one of the following proteins: phospho-protein kinase RNA-like ER kinase (phospho-PERK), PERK (Cell Signaling Technology, USA), C/EBP homologous protein (CHOP), glucose-regulated protein 78 (GRP78), cytochrome c (Santa Cruz Biotechnology, USA), peroxisome proliferator-activated receptor γ-coactivator-1α (PGC-1α), phospho-AMP-activated protein kinase (AMPK), AMPK, or β-actin (Calbiochem, Germany). The membrane was then washed with TBST and exposed to secondary antibodies for 60 min. ECL (Genekhan Scientific Corp.,USA) was used for visualization, and SigmaGel (Jandel Scientific Corp., Germany) was used to control for relative band intensity. The intensity of target band signals from every individual blot was normalized by β-actin band intensity, which was measured from similar membranes after treatment with restored stripping buffer. These signal intensities were averaged in each group and were calculated into arbitrary units. β-actin was determined by comparing similar membranes with target antibodies.

### Statistical analysis

Data are reported as means±SD, and SPSS 10.0 (SPSS Inc., USA) was used for statistical analysis. To investigate differences between groups after 12 weeks of treatment, one-way ANOVA (western blot and heart weight data) and two-way ANOVA (body weight and food consumption data) were conducted. Tukey's test was used for *post hoc* analysis. The level of statistical significance was set as α=0.05.

## Results

### Body weight and food intake

**Figure 1.**
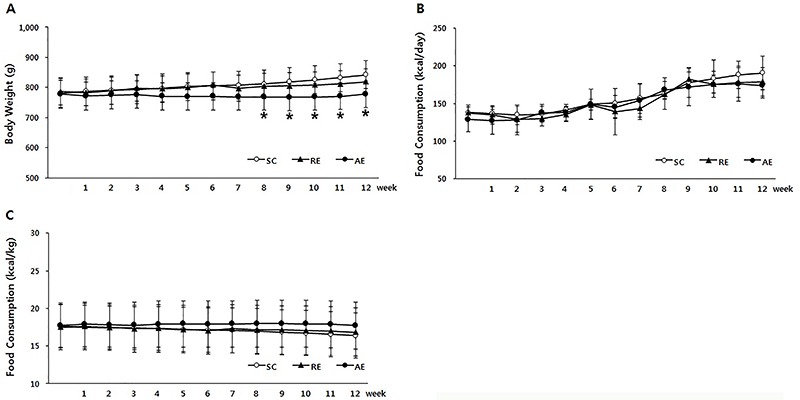
Changes in body weight (*A*), daily caloric intake (*B*), and daily caloric intake per kg of body weight (*C*) during 12 weeks of aerobic exercise (AE) and resistance exercise (RE). Data are reported as means±SD. *P<0.05 compared to the sedentary control (SC) group (two-way ANOVA).

The body weight of obese rats in the SC group gradually increased over the 12 weeks of the experiment, and that of those in the RE group demonstrated similar increases. However, body weight showed significantly lower values in the AE group than the SC group (P<0.05) from week 8 (Figure 1A). Although food intake increased progressively over the 12 weeks, no significant difference among the groups was noted in this respect (Figure 1B). Food intake per kg of body weight also showed no significant difference among the groups (Figure 1C).

### Heart weight and intraperitoneal fat

**Figure 2.**
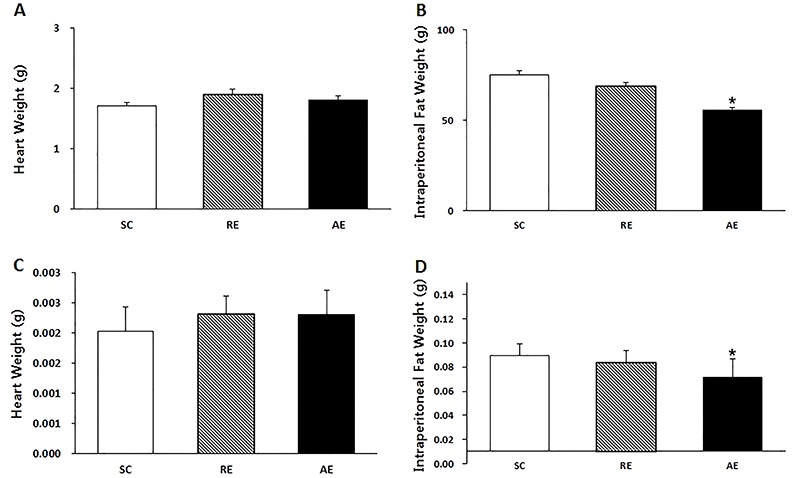
Comparison of heart weight (*A*), intraperitoneal fat weight (*B*), heart weight per 1 kg of body weight (*C*), and intraperitoneal fat weight per kg of body weight (*D*) after 12 weeks of aerobic exercise (AE) and resistance exercise (RE). Data are reported as means±SD. *P<0.05 compared to the sedentary control (SC) group (one-way ANOVA).

The heart weight of the middle-aged rats with high-fat diet-induced obesity did not significantly differ among the groups after 12 weeks of exercise training (Figure 2A). The amount of intraperitoneal fat carried by the rats was not affected by 12 weeks of RE; however, it was significantly reduced by AE for the same period compared to the SC group (P<0.05) (Figure 2B). Additionally, heart weight per 1 kg of body weight (Figure 2C) and intraperitoneal fat weight per kg of body weight (Figure 2D) showed similar results with absolute values.

### Mitochondrial enzymes

**Figure 3.**
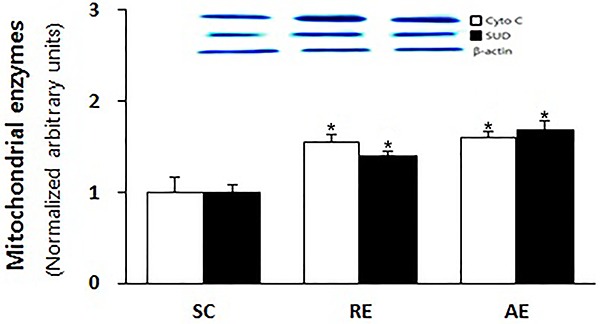
Mitochondrial enzyme levels within cardiac muscle after 12 weeks of aerobic exercise (AE) and resistance exercise (RE). Cyto C: cytochrome C; SUD, succinate dehydrogenase. Data are reported as means±SD. *P<0.05 compared to the sedentary control (SC) group (one-way ANOVA).

To observe changes in cardiac muscle mitochondrial biogenesis, levels of the marker proteins cytochrome c and succinate dehydrogenase were measured. Twelve weeks of ladder climbing or treadmill running exercise significantly increased mitochondrial enzyme levels in the cardiac muscle of middle-aged obese rats (P<0.05). However, no significant difference was noted according to exercise type (Figure 3).

### ER stress markers

**Figure 4.**
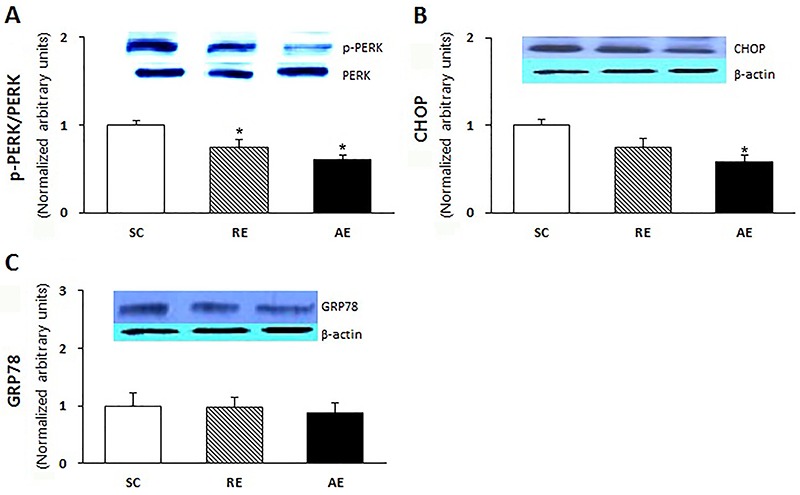
Endoplasmic reticulum stress markers, phospho-PERK/PERK ratio (*A*), CHOP protein level (*B*), and GRP78 protein level (*C*) within the myocardium after 12 weeks of aerobic exercise (AE) and resistance exercise (RE). Data are reported as means±SD. *P<0.05 compared to the sedentary control (SC) group (one-way ANOVA).

In order to measure ER stress in cardiac muscle, the phospho-PERK/PERK ratio and level of CHOP protein, indicative of myocardial damage, as well as levels of GRP78, a myoprotective protein, were measured, among other ER stress proteins. Significant decreases (P<0.05) in the phospho-PERK/PERK ratio were noted in both exercise groups compared to the SC group (Figure 4A). Although the level of CHOP protein was lower in the exercise groups than the SC group, only the AE group differed significantly in this respect (P<0.05) (Figure 4B and C). Levels of the myoprotective protein GRP78 did not differ significantly between the groups, i.e., they were not affected by exercise training (Figure 4C).

### PGC-1α and phospho-AMPK/total AMPK ratio

**Figure 5.**
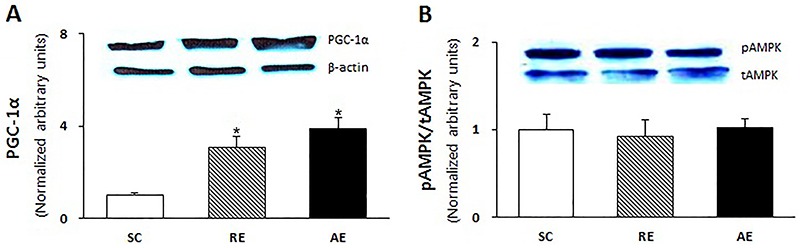
PGC-1α protein level (*A*) and phospho-AMPK/total AMPK protein ratio (*B*) within the myocardium after 12 weeks of aerobic exercise (AE) and resistance exercise (RE). Data are reported as means±SD. *P<0.05 compared to the sedentary control (SC) group (one-way ANOVA).

In order to investigate the effects of 12 weeks of ladder climbing or treadmill running exercise on mitochondrial biogenesis in the myocardium of obese middle-aged rats, the level of PGC-1α protein, an upstream modulator, was measured. In rats that underwent exercise, myocardial PGC-1α levels were significantly higher (P<0.05); however, no significant difference was noted between the two exercise types (Figure 5A). To investigate the effects of 12 weeks of exercise training on AMPK activity within the myocardium of obese middle-aged rats, phospho-AMPK and total AMPK were quantified and the resulting ratio was analyzed. AMPK activity did not change due to the 12 weeks of exercise training and did not differ according to exercise type (Figure 5B).

## Discussion

The ER is an important organelle involved in the regulation of intracellular calcium and protein folding. During ER stress, the unfolded protein response (UPR) activates the following protective mechanisms: prevention of protein translation, decreased introduction of new proteins, enhancement of the protein folding ability of the ER, removal of misfolded proteins, and apoptosis to eliminate damaged cells (19). Chronic ER stress causes oxidative stress and inflammatory responses (20), and obesity is known to increase ER stress in metabolically active tissues (12). The activity of PERK, phospho-inositol-requiring enzyme 1α (phospho-IRE1α), and c-Jun N-terminal kinase (JNK) in the fat and liver tissues of mice kept on a high-fat diet has been found to be significantly higher than that in lean animals (21). Moreover, *ob*/*ob* mice exhibit elevated ER stress, JNK activity, and PERK and phospho-IRE1α levels in adipose and hepatic tissues (22).

It has been reported that long-term exercise training exerts positive influences on myocardial ER stress. González et al. (23) demonstrated that 3 months of endurance training significantly increases GRP78 levels in skeletal muscles, including the soleus and extensor digitorum longus. GRP78 and GRP94, which are ER translocation and polypeptide-chain-folding molecular chaperones (24), act to protect myocardial cells against damage from myocardial ischemia (25) and cell death is decreased by activation of these proteins (26). However, Bozi et al. (27) identified GRP78 as a typical UPR marker that acts to maintain ER homeostasis in cardiac tissue, with its level increasing significantly in rats with myocardial infarction and decreasing significantly after AE training. In the present study, no significant change in GRP78 levels in cardiac tissue was noted after 12 weeks of exercise training. Although GRP78 inhibits ER transmembrane proteins in the unstressed state, it separates from these proteins under ER stress and induces the UPR (28). The current study indicated that exercise training does not significantly affect GRP78, which is associated with ER stress.

In contrast, in the present study, phosphorylation of PERK and expression of CHOP protein, which are markers of ER stress (28), decreased significantly after exercise training. A tendency for decreased CHOP expression was noted in both exercise groups and significantly lower levels were observed in the AE group (P<0.05). Consistent with our findings, increased phosphorylation of PERK and eukaryotic translation initiation factor 2α (eIF2α) has been reported in animals administered a high-fat diet. In addition, endurance exercise has been shown to result in decreased expression of PERK and eIF2α in skeletal muscle (29). In the present work, we found long-term exercise training to result in inhibition of ER stress marker expression within the myocardium of middle-aged obese rats, and established that AE is more effective than RE in this respect. Nonetheless, in the RE group, phosphorylation of PERK also decreased significantly and CHOP expression was reduced. Furthermore, our previous work has shown that 8 weeks of resistance training decreases PERK phosphorylation and CHOP levels in the myocardium (12); thus, such exercise does appear to affect these factors. It seems likely that AE was particularly effective because the significant decreases in body weight and intraperitoneal fat observed in rats of this group led to a decrease in the degree of obesity, and obesity is known to be responsible for ER stress (30). However, since our findings cannot demonstrate a causal relationship, this needs to be investigated further in future studies.

Regarding mitochondrial biogenesis, transcription factors and cofactors that regulate oxidative phosphorylation, heme biosynthesis, the oxidative phosphorylation system, and mtDNA transcription and replication have been identified previously (31). These include nuclear respiratory factor 1 and 2 (NRF-1 and NRF-2) (32), estrogen-related receptor α (ERRα), and the transcriptional cofactors PGC-1α, PGC-1β, and PGC-1-related coactivator (33). Given this, endurance training is thought to be effective in promoting skeletal muscle mitochondrial biogenesis, increasing the number and activity of mitochondria (34). Multiple studies have also reported that resistance training can increase PGC-1α expression (35,36). In the current work, PGC-1α levels were found to be significantly increased in both the RE and AE groups, as were levels of markers of mitochondrial biogenesis. Activation of mitochondrial biogenesis in the myocardium due to resistance training has also been confirmed in one of our previous studies (12), and long, low-intensity resistance training resulting in fatigue is thought to promote mitochondrial biogenesis (37). Moreover, the present investigation confirmed that RE and AE are similar in terms of the relationship between mitochondrial biogenesis and ER stress, a finding that is supported by a previous study, in which changes in PERK were shown to influence the mitochondria-associated ER membrane (20).

According to previous research, high-intensity exercise above the lactate threshold (9) or extended exercise (38) is required to induce increases in skeletal muscle mitochondrial biogenesis through elevated expression of PGC-1α protein. In a study similar to that described here, Kim et al. (39) analyzed the influence of 5 weeks of low- or high-intensity endurance training on mitochondrial biogenesis and ER stress in the skeletal muscle of rats. Although no significant differences in body weight and fat mass were noted, they found that only high-intensity exercise resulted in increased mitochondrial biogenesis and decreased ER stress. In the current work, no significant difference was observed between the RE and SC groups in terms of body weight and intraperitoneal fat. Although low-intensity RE might not result in significant weight loss, it can still lead to activation of mitochondrial biogenesis in the myocardium.

To further investigate changes in mitochondrial biogenesis, we aimed to analyze differences in AMPK activity according to energy consumption in each exercise group using the ratio of phospho-AMPK to total AMPK; however, no significant difference was noted between the groups. This lack of a difference in AMPK activity, an important regulator of mitochondrial biogenesis during exercise (40), may have been due to the fact that the samples were collected 48 h after completion of the exercise programs, by which time the activation of AMPK would have come to an end due to its short half-life. Future studies on AMPK activity should consider the cardiac tissue sampling time due to AMPK’s short half-life.

In this study, we analyzed the effects of long-term exercise training on ER stress and mitochondrial biogenesis in the myocardium of middle-aged rats with high-fat-diet-induced obesity. We observed increased mitochondrial biogenesis and decreased ER stress after RE (ladder climbing) and AE (treadmill running). Therefore, aerobic training resulting in weight loss, as well as resistance training that does not necessarily lead to weight loss, can contribute to the prevention of obesity-associated decreases in cardiac function in this experimental model.
